# Athletes' and Coaches' Attitudes Toward Protective Headgear as Concussion and Head Injury Prevention: A Scoping Review

**DOI:** 10.3389/fspor.2021.680773

**Published:** 2021-05-25

**Authors:** Anne Tjønndal, Frida Austmo Wågan

**Affiliations:** Faculty of Social Sciences, Nord University, Bodø, Norway

**Keywords:** helmet, mouthguard, headguard, sport and injury prevention, sports related concussion, protective headgear and attitudes

## Abstract

The purpose of this article is to map existing research literature on athletes and coaches' attitudes toward protective headgear in sport in relation to concussion and head injury prevention, and to identify and analyse knowledge gaps in the field. A scoping review was conducted in three databases; PubMed, Scopus, SportDiscus, and reference lists were searched to identify relevant grey literature. This process lead to an in-depth analysis of 18 peer-reviewed journal articles. Of the 18 studies identified, the majority focused on athletes (*n* = 14), only two studies focused on coaches, and two studies included a sample of both athletes and coaches. The findings in this scoping review suggests that there is a discrepancy between attitudes and beliefs about the protective effects of headgear, athletes' behaviour as far as wearing protective headgear, and coaches' behaviour in terms of recommending use of protective headgear to their athletes. The majority of athletes in most of the reviewed literature believed that headgear had protective effects against concussion and other head injuries, however relatively few athletes report wearing this protective headgear unless it was mandatory by competition rules.

## Introduction

Concussion is a commonly reported injury among adult and youth athletes around the globe. Consequently, sport related concussion has become an issue of concern in both contact and non-contact sports at the elite and recreational level (Scher et al., 2017; Malcolm, 2019; Ventresca and McDonald, 2020). Clinically, concussion is known to affect an athlete's memory, reaction time (Covassin et al., 2008) and balance (McCrea et al., 2003). A previous concussion may also mean athletes being prone to subsequent concussions (Covassin et al., 2008; King et al., 2014), which could result in mild cognitive impairment (Guskiewicz et al., 2005), depression (Guskiewicz et al., 2007) or chronic traumatic encephalopathy (CTE) (McKee et al., 2009).

Due to the clinical effects of concussion and the risk of head trauma in many sports, more concerns have been raised in the last decade about sport-related concussion. There has also been an increase in the amount of research, media coverage, educational and policy interventions addressing the phenomenon (Sarmiento et al., 2017). In particular, a substantial body of biomedical research on sport-related concussion has been conducted in different sports, such as horse racing (Mattacola et al., 2017), ice hockey (Tegner and Lorentzon, 1996), rugby (Gardner et al., 2015), combat sports (Follmer et al., 2020), volleyball (Meeuwisse et al., 2017), and football (Leung et al., 2017).

Biomedical and social scientific studies of sport-related concussion include explorations of protective measures, such as programmes to educate coaches and athletes about the symptoms, rule changes in sport to minimise the risk of impacts to the head and the use of protective equipment like headguards and mouthguards. Some studies suggest that headguards, helmets and mouthguards may offer some protection against concussion and other head injuries (King et al., 2014; O'Sullivan and Fife, 2016; Tjønndal et al., 2021). Mouthguards are the most controversial in terms of protecting against injuries other than orodental ones (McCrory, 2001; Tiryaki et al., 2017). Research also indicates that athletes' use of protective headgear varies greatly in different sporting contexts (Lehl, 2005).

Sarmiento et al. (2017) argue that attitudes strongly influence behaviour when it comes to athletes' use of protective headgear and other preventive measures. In their study, the authors explored concussion education and knowledge in American youth and high school sports. In our article we focus on athletes' and coaches' attitudes toward protective headgear as concussion and head injury prevention in sport. To our knowledge, no scoping reviews have examined this topic with an international lens. The purpose of this scoping review is therefore to: (1) summarise and map the existing research literature on athletes' and coaches' attitudes toward protective headgear in sport, (2) identify and analyse knowledge gaps in the field and (3) propose an agenda for future research based on the reviewed literature.

## Materials and Methods

According to Munn et al. (2018), scoping reviews often act a precursor to systematic reviews. The purpose of a scoping review is to map the available knowledge in a particular field, examine how research is conducted on a certain topic and identify and analyse knowledge gaps. As scoping reviews do not aim to synthesise knowledge in order to answer a specific research question, but rather provide an overview of a research field and map the “state of the art” in that field, they do not usually include methodological processes such as an assessment of methodological limitations or risk of bias (in medical studies) (Peters et al., 2015). In other words, scoping reviews are appropriate when the body of literature is relatively new and the goal is to map the current findings, identify knowledge gaps and investigate how research is conducted on a specific topic (Munn et al., 2018; Peters et al., 2020).

In this article we use a scoping review methodology to gain an overview of the current research on athletes' and coaches' attitudes toward headgear as a protective measure to minimise the risk of concussion and other head injuries in sport. In the following, we present our search strategy, the procedure of the review and our analysis of the identified literature.

### Data Sources and Search Strategy

Three electronic databases - PubMed, Scopus and SportDiscus - were searched to identify the relevant studies for review. These databases were selected because they collectively provide insights into biomedical, behavioural and social science research on head injuries and the use of protective headgear in sport. The following four searches were conducted in all three databases: (1) “sport” AND “helmet” OR “headguard” OR “headgear” AND “perceptions” OR “attitudes,” (2) “headgear” AND “athlete” OR “coach” AND “attitudes,” (3) “concussion” AND “knowledge” AND “athlete,” (4) “concussion” AND “knowledge” AND “coach.” The search was conducted in February 2021 with no limitation on publication dates in order to yield all possible articles on the subject. Additionally, searches of grey literature were performed to identify other studies published in English that may not have been identified through the database searches. The grey literature search included an examination of the reference list material in the identified studies. Table 1 identifies the specific search strategies used for each database, search engine and number of hits.

**Table 1:** Search strategies and initial number of hits.

	**Sport, helmet/headguard/ headgear AND perceptions/ attitudes**	**Headgear AND athlete/coach AND attitudes**	**Concussion AND knowledge AND athlete**	**Concussion AND knowledge AND coach**	**Total**
PubMed	*n* = 86	*n* = 14	*n* = 501	*n* = 116	*n* = 717
Scopus	*n* = 52	*n* = 2	*n* = 326	*n* = 79	*n* = 459
SportDiscus	*n* = 13	*n* = 2	*n* = 92	*n* = 48	*n* = 155
					*n* = 1331

All the identified research literature describing the attitudes toward protective headgear in sport, concussion knowledge, the use of protective headgear in sport and the perceived risk of concussion and head injuries in athletes and/or coaches at all levels was included in the preliminary analyses. A number of inclusion and exclusion criteria were used to check the eligibility of titles, abstracts and full-text articles. The inclusion criteria stipulated that the studies included in the review should:

1. be reported in peer-reviewed journals and published in English.
2. be original empirical studies.
3. include a sample and population of interest consisting of athletes and/or coaches.
4. focus on attitudes toward protective headgear and concussion/head injury prevention.

The rationale for these criteria was a need to identify international academic publications on attitudes toward headguards to prevent head injuries in sport.

### Procedure of the Scoping Review

The studies for final review were identified in three steps. First, preliminary searches for titles, abstracts and keywords were undertaken to identify articles that fitted the inclusion criteria. Second, full-text articles were assessed for eligibility. At this stage, several articles were removed from the review due to duplication with a previously identified article across the three databases or only describing concussion knowledge/behaviour/attitudes. Thirdly, questions about the inclusion and exclusion of articles were discussed and resolved collectively by the two authors.

In the first step, 1,331 articles were identified in the initial searches of the databases (see Table 1). The screening of the initial number of hits in the three databases revealed that many of the identified articles did not match the purpose of our literature review or fulfil the inclusion criteria. As illustrated in Table 1, we identified a large number of hits on the topic of concussion knowledge among athletes and coaches, although only a few of these studies took attitudes toward headgear into consideration. After reviewing the initial hits according to the inclusion criteria, 843 studies were excluded, leaving us with 488 relevant studies (see Table 2).

**Table 2:** Studies identified as relevant to the initial inclusion criteria in the three databases before removing duplicates.

	**Sport, helmet/headguard/ headgear AND perceptions/ attitudes**	**Headgear AND athlete/coach AND attitudes**	**Concussion AND knowledge AND athlete**	**Concussion AND knowledge AND coach**	**Total**
PubMed	*n* = 9	*n* = 7	*n* = 135	*n* = 6	*n* = 157
Scopus	*n* = 10	*n* = 1	*n* = 131	*n* = 79	*n* = 221
SportDiscus	*n* = 4	*n* = 2	*n* = 63	*n* = 41	*n* = 110
					*n* = 488

After removing the duplicates (*n* = 1,139), we were left with 192 studies in which the abstract met the initial inclusion criteria of attitudes toward or knowledge about head injuries/concussion or protective headgear amongst coaches or athletes. Eleven articles were further identified as relevant in the grey literature searches after examining the reference list material in the identified studies, thus leaving us with a total of 203 studies.

**Figure 1:**
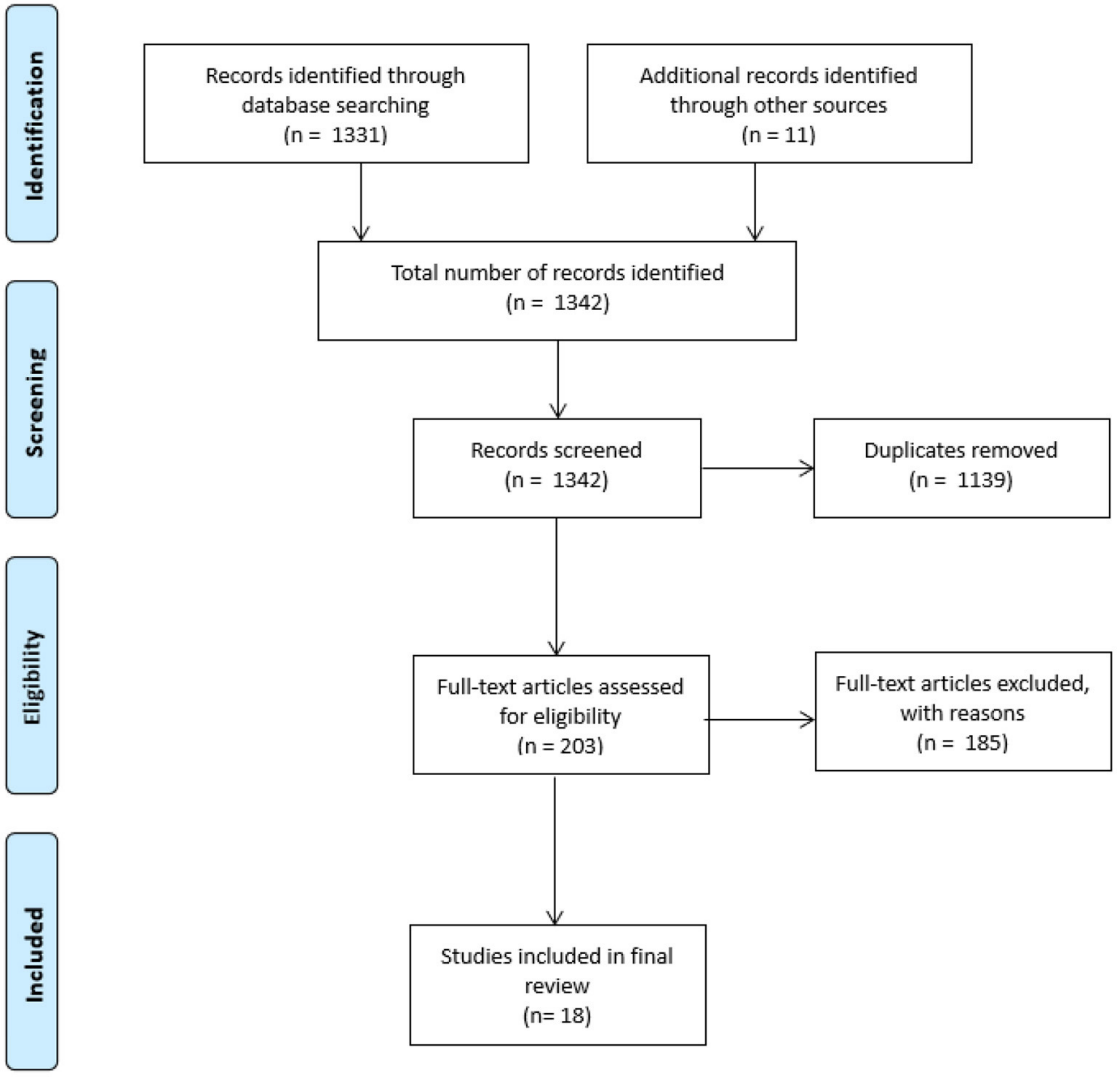
Flow chart of the scoping review process.

In the second step of assessing the full-texts for eligibility, we identified 185 studies that examined concussion knowledge but did not include attitudes toward protective headgear amongst parents, athletes, coaches, health personnel, teachers or other stakeholders. These were excluded from our review as they did not meet the inclusion criteria. Twenty studies were then considered according to method and study populations. A further two studies were excluded in this step because the sample did not include coaches or athletes, thereby leaving us with 18 studies for the final review. The procedure of the scoping review is illustrated in Figure 1.

## Results

The preliminary literature searches revealed a substantial body of research on concussion knowledge amongst athletes and/or coaches, with 203 identified studies in the procedure of the scoping review. When limiting the search to studies exploring knowledge about or attitudes toward protective headgear in relation to concussion or other head injuries, the number of studies meeting the inclusion criteria was limited to 18 (see Table 3).

**Table 3:** Results of the scoping review.

**References**	**Aim**	**Sample**	**Method and measures**	**Findings**
Pettersen (2002)	Examine the attitudes of players and coaches toward the use of protective headgear, particularly with respect to the prevention of concussion.	63 Canadian male and female rugby players and nine coaches from four different Canadian teams, each representing a different level of play (high school, university, community club, national).	a. Survey to address the athletes' attitudes toward headgear and its use. b. Survey or telephone interview (structured) to address coaches' views of team policies and their personal opinions about the use of headgear to prevent concussion.	62% of players and 33% of coaches believed that wearing headgear could prevent concussion. Despite the players' beliefs that headgear offers protection against concussion, only a minority reported wearing it (27%) and few (24%) felt that its use should be compulsory. Common reasons for not wearing headgear were “its use is not mandatory,” “it is uncomfortable,” and “it costs too much.”
Braham et al. (2004)	Examine community football players' attitudes toward protective equipment.	301 Australian community footballers.	Self-report questionnaire.	73.6% of the players reported wearing mouthguards during the previous playing season, compared with only 2.1% wearing headgear. The most common reasons for not wearing headgear and mouthguards (in non-users) were: “I don't like wearing it” (headgear: 44.8%, mouthguards: 30.6%) and “It is too uncomfortable” (headgear: 40.7%, mouthguards: 45.8%).
Braham and Finch (2004)	Assess the extent to which players use the protective equipment they are allocated to wear.	23 senior and junior teams from football clubs in Victoria, Australia, with a total of 301 players (aged 20–22 years).	Observational study as part of the Australian Football Injury Prevention Project (AFIPP). Club-based primary data collectors (PDCs) were formally trained in the same protocol to observe and record whether or not each player wore headgear or a mouthguard during each training session and game throughout the season.	Mouthgard use was higher than headgear use, with the highest usage for both being measured during games rather than training. Although many players use mouthguards, particularly in games, most do not wear headgear.
Finch et al. (2001)	Determine the attitudes of schoolboy rugby union players toward protective headgear.	140 rugby union players from 10 randomly selected school teams in Sydney (aged 14–16).	Self-report questionnaire.	Some form of protective equipment was always worn by 76.1% of players, where 93.6% reported using a mouthguard and 79.3% a helmet/headgear. The two most important reasons for wearing headgear were related to safety concerns. Of the players who wore headgear during the 1999 season, 67% said that they played more confidently when wearing it, but 63% said that their head was hotter with it. Few players reported that their head was uncomfortable (15%) or that it was hard to communicate (3%) when wearing headgear. The main reasons for not wearing headgear were related to design features: uncomfortable (61%) and hot (57%).
Jeffries et al. (2020)	To evaluate the concussion-prevention strategies used in National Collegiate Athletic Association Division I and Division II women's soccer and identify the beliefs of certified ATs regarding mechanisms for preventing concussion.	A total of 223 women's soccer team athletic trainers employed at Division I or II universities.	Semi-structured online survey (self-report questionnaire).	70% of collegiate women's soccer athletic trainers (ATs) believed that cervical-strengthening programmes would help to prevent concussion, but only 17% currently used such programmes. 8.7% believed that soccer headgear prevented concussion, and 20% believed that mouthguards prevented concussion. Education in proper soccer techniques was reported by 151 (69.59%) respondents, and seventy-eight (35.49%) reported that their players wore headgear. There was a mismatch between the clinical beliefs of ATs and the implementation of concussion-prevention strategies amongst women's collegiate soccer players.
Kahanov et al. (2005)	Determine American collegiate rugby players' perceptions of the relationship between rugby union soft-shelled headgear and its effectiveness for the prevention of concussive head injuries.	131 male rugby union participants from eight university teams in the United States.	Online survey (self-report questionnaire).	Player position and years of experience played a role in the number of concussions and use of protective headgear. Seventy-six different athletes reported a concussion while playing, with the majority (51%) not wearing headgear. Athletes who wore headgear experienced 24% of the concussions, compared to 76% of those who did not wear headgear. The incidence of concussion and its severity was perceived as less severe in the group wearing headgear. The general perception of those individuals polled as to the effectiveness of headgear in reducing head injuries was positive.
Lehl (2005)	Evaluate sport coaches' perceptions, knowledge and experience of orofacial injuries and their prevention.	40 coaches from the Sports Department, Chandigarh (involved in training of youth at high school, college and university level).	Self-report questionnaire.	The coaches considered helmets to be the most common protective device, followed by mouthguard and facemask. About 58% observed that boxing was associated with orofacial injuries. Protective devices were deemed compulsory by 68% in this event. About 45% saw over five injuries in the last year, mostly soft tissue facial injuries (47%) and tooth loss (33%). Most injuries were in hockey and 32% were due to hits by ball, stick or related hard objects. About 82% were related to the non-use of protective devices. The majority of coaches considered that orofacial devices should be made more popular among sportspersons for their safety, while 28% felt they reduced efficiency.
Pratt et al. (2019)	Assess the attitudes of mountain bikers to the use of protective equipment and quantify the use of such equipment.	263 Riders competing in NZ Enduro Crown races across New Zealand (aged 18–65).	Prospective cohort study using an online questionnaire.	Equipment use was similar in racing and non-racing settings, where 30–35% reported always wearing a helmet, ~30% sometimes wearing a helmet and ~30% never wearing a helmet. 55% had experienced an injury requiring a week or more off work. Perceptions of the benefits, costs, cues, comfort and potential injury severity proved to be well correlated with the decision to use equipment, while harm, danger and exposure to media influences did not.
Ross et al. (2010)	Examine usage rates and ostacles to the use of protective equipment in roughstock rodeo athletes.	189 roughstock rodeo athletes (aged 18–36).	Self-report questionnaire.	69% never wore a helmet in competitions. Obstacles to helmet-use were a negative effect on performance and sport persona. 58% always used a mouthpiece and 21% did not. Obstacles were discomfort and forgetfulness. Reported injury rate was high (only 7.5% reported no injuries during their careers), with users noting fewer injuries to head and ribs than non-users. Riders agreed that protective equipment prevented injury to the head, ribs and mouth. However, equipment usage rates varied widely by type and seemed to be underutilised because the equipment affected performance, was uncomfortable and “not cowboy.”
Ruedl et al. (2012a)	Compare attitudes toward the use of ski helmets in wearers and non-wearers.	924 people (52% men and 48% women participating in sports programmes at the University Sports Institute Innsbruck/Austria (mean age 31 years).	Structured quantitative interviews.	In total, 65% of participants wore a helmet for their preferred winter sport activity, while more than 80% of wearers and non-wearers agreed that helmets protected against head injuries. The non-use of helmets increased with age and decreased with increasing skill level. In addition, non-use was associated with subjective disadvantages, safety awareness, comfort/style and risk compensation.
Ruedl et al. (2012b)	Evaluate whether risk taking behaviour is associated with the personality trait of sensation seeking (SS) in alpine skiing and snowboarding.	683 people (36% males and 64% females) completed an online survey about attitudes toward and use of protective gear.	Online self-report questionnaire, including the German version of the sensation- seeking scale form V (40-item forced-choice questionnaire assessing the sensation seeking construct as a total score).	Risky behaviour increased with male gender (OR: 2.7), with an age < 25 years (OR: 1.6), with skiing (OR: 1.3), higher skill levels (OR: 5.7) and a mean skiing time > 28 days per season (OR: 2.2). The use of ski helmets was not found to be predictive of riskier behaviour (*p* > 0.05). In addition, self-reported risk compensation in helmet wearers increased with age < 25 years (OR: 2.2), a higher skill level (OR: 2.5) and a mean skiing time > 28 days per season (OR: 2.1). The personality trait sensation seeking and not wearing a ski helmet appears to be associated with riskier behaviour on the ski slopes.
Taylor et al. (2005)	Examine the use of protective headgear by surfers, their perceptions of its usefulness and obstacles to its use.	646 surfboard riders at eight popular surfing beaches in Victoria, Australia.	Researcher-administered questionnaire.	Only 38% surfers considered the risk of head injury while surfing as moderate or high, and only 1.9% reported the routine use of headgear. The surfers were more likely to believe that there was a greater risk of head injury in other sports and physical activities. Although 73% thought that surfers who wore headgear were less likely to be injured, only 62.1% reported that headgear restricted surfing performance and that they would rather surf without it. The main reasons for not wearing headgear were “no need,” discomfort, claustrophobia and effects on the senses and balance.
Vriend et al. (2018)	Evaluate the effects of exposure to a nationwide intervention on relevant determinants of helmet use and helmet use in Dutch recreational skiers and snowboarders (DRSS).	363 Dutch recreational skiers and snowboarders (DRSS) (aged 18–65).	A prospective single-cohort study was conducted to evaluate the impact of intervention exposure on determinants of helmet use (i.e., knowledge about head injury risk and preventive measures, risk perception, attitudes to head injury risk and helmet use and intention to wear a helmet) and self-reported helmet use.	Overall, no significant associations were found between intervention exposure and the determinants of helmet use. However, sub-group analyses revealed intervention effects on risk perception and knowledge in specific sub-populations. Intervention exposure had a significant, positive effect on helmet use in DRSS (β = 0.23; 95% CI 0.017–0.44). Sub-group analyses revealed that this effect was found in: (1) skiers, (2) female DRSS, (3) young skiers and (4) intermediate skiers. Overall, intervention reach was 28.1%, with differences found between skiers and snowboarders.
Tiryaki et al. (2017)	Determine the prevalence of dental injuries and investigate the awareness of the use of mouthguards among basketball players and coaches.	53 basketball coaches (18–69 years) and 351 basketball players (12–38 years).	Self-report questionnaire.	124 players (35% of the total sample) had experienced oral injuries, including soft tissue lacerations (80.6%), fractures (17.7%) and avulsions (1.6%). Although the players had sustained dental injuries, 95% of them found mouthguards to be protective, and only 6.3% actually used them. The rate of mouthguard use among players who had experienced an oral injury was significantly higher than that among players with no history of injury. Although 98% of the coaches believed that mouthguards could prevent dental injuries, only 47% of them recommended their use to their players. The most common reasons for not using a mouthguard were discomfort (37.7%) and difficulty in breathing (7.3%) or talking (6.4%).
Inness and Morgan (2015)	Examine risk perception, mitigation and risk factors for injuries and falls in UK polo players.	112 UK polo players.	Retrospective telephone questionnaire.	Injuries (commonly to a shoulder or wrist) requiring a hospital visit were sustained by 17.3% of players, where falls and higher self-assessed fitness levels increased the risk and use of wrist supports and gym exercise reduced it. Falls were reported by 58% players, and women were at less risk than men. Aiming for a better handicap increased the risk of falls. Pre-season rider and horse training were also risk factors. Helmets are compulsory, but players reported that safety certification was not their most important criterion for helmet selection; 49.4% chose appearance.
Finch et al. (2003)	Explore the attitudes of community-level Australian football players toward protective headgear and the risk of head injury.	70 players from four purposefully chosen clubs in metropolitan Melbourne (ages 15–51).	Self-report questionnaire.	Almost all the players (91.4%) reported that they did not wear protective headgear. Non-headgear users said that headgear was too uncomfortable (47.4%) and they didn't like it (42.1%). However, 80.0% of non-users said they would wear it if it prevented injury. As a group, the players perceived the risk of head injury in Australian Rules football to be low to moderate when compared to other sports and activities, and the players considered rugby, boxing and driving a car to be associated with a higher risk of head injury than Australian Rules football.
Dean and Bundon (2020)	Explore surfers' perceptions of and attitudes toward protective headgear, and specifically explore why so few surfers wear protective headgear.	12 experienced surfers from Canada.	A qualitative methodology including both participant observations and qualitative interviews were used (12 experienced surfers from the West Coast of Canada were interviewed and over 30 h of participant observations were collected).	The surfers reported not wearing protective headgear for four main reasons: (1) that protective headgear is uncomfortable and could hinder performance, (2) the perception that protective headgear is only for other surfers, (3) the belief that surfing is not a high-risk sport and (4) for aesthetic reasons and/or the appearance of protective headgear.
Provance et al. (2012)	Assess the influencing factors in youth who do not wear a helmet while skiing or snowboarding.	206 children/adolescents (aged 6–17) and their parents were enrolled in the study.	Self-report questionnaire.	83% reported that they wore a ski/snowboard helmet. There was a significant relationship between parental helmet use and child helmet use. Of the 171 children/adolescents who reported wearing a helmet, 124 (72.5%) reported that it protected them in an accident. Of all the helmet-wearers, 87.7% said that safety was the main reason for wearing one. The most common reason for not wearing a ski/snowboard helmet was comfort (48.6% of all non-helmet users).

Of the 18 articles that were included in the final review: three focused on knowledge about and awareness of the risk of injuries to the head and injury prevention strategies (Lehl, 2005; Inness and Morgan, 2015; Jeffries et al., 2020), five examined perceptions of the risk of head injury and attitudes toward the use of protective headgear (Kahanov et al., 2005; Taylor et al., 2005; Provance et al., 2012; Tiryaki et al., 2017; Vriend et al., 2018), seven studies only examined athletes' attitudes toward using protective headgear in sport (Finch et al., 2001, 2003; Pettersen, 2002; Braham et al., 2004; Ruedl et al., 2012a,b; Pratt et al., 2019), two examined athletes' experiences of the obstacles to wearing protective headgear in training and competitions (Ross et al., 2010; Dean and Bundon, 2020) and one only examined the use of protective headgear in one single season (Braham and Finch, 2004). Finally, four studies did not include any measurement of the athletes' use of protective headgear, but the remaining 14 of the 18 studies did.

The reviewed studies examined a wide range of sports, including polo (*n* = 1), roughstock rodeo (*n* = 1), downhill skiing (*n* = 4), rugby (*n* = 2), surfing (*n* = 2), basketball (*n* = 1), Australian football (*n* = 5), different recreational sports including hockey, basketball, volleyball, cricket, football and martial arts (*n* = 1) and mountain biking (*n* = 1). Fourteen of the studies focused on athletes, two on coaches (Lehl, 2005; Jeffries et al., 2020) and two included a sample of athletes and coaches (Pettersen, 2002; Tiryaki et al., 2017). The study populations varied in age from 6 years (Provance et al., 2012) to 65 years (Pratt et al., 2019).

### Athletes' Use of and Attitudes Toward Protective Headgear in Sport

Several studies reported on athletes' use of protective headgear (headgear behaviour). The reported use of protective headgear varied greatly from ~2% of the sample in Taylor et al.'s (2005) study of surfers and Braham et al.'s (2004) study of community football, to 80–90% of the sample in Finch et al.'s (2003) rugby study and Provance et al.'s (2012) study of skiing and snowboarding. Some of the variations relating to the use of headgear could be due to differences in the competition rules of different sports, especially if wearing a helmet or protective headgear is compulsory, optional or in some cases (as in men's boxing) forbidden. The ongoing debate about whether protective headgear should be compulsory in rugby (Frizzell et al., 2018) and skiing/snowboarding (Alsop et al., 2013), or the studies showing a reduced risk of head injuries when skiers and snowboarders (Russell et al., 2010; Haider et al., 2012) or rugby players (Frizzell et al., 2018) wear helmets, could also explain this variation. However, when it comes to athletes' attitudes toward wearing protective headgear, the variation in perceptions is considerably smaller. In studies reporting on athletes' attitudes in percentages, we identified a variation between 62% (Pettersen, 2002) and 87.7% (Provance et al., 2012) in the athletes included in the samples who believed that wearing headgear protected against concussion and head injuries (see Table 4).

**Table 4:** Summary of studies reporting on athlete/coach attitudes and behaviour in percentages.

**References**	**Athlete attitudes**	**Coach attitudes**	**Athlete behaviour**	**Coach behaviour**
Pettersen (2002)	62% believed that wearing headgear could prevent concussion.	33% believed that wearing headgear could prevent concussion.	27% reported wearing headgear.	None
Braham et al. (2004)	44.8% reported that they did not like wearing headguards, 40.7% reported that wearing a helmet was uncomfortable.	None	73.6% reported wearing mouthguards, 2.1% reported wearing headgear.	None
Finch et al. (2001)	67% reported playing more confidently when wearing headgear, 63% reported that the headguard made their head hot.	None	93.6% reported wearing mouthguards and 79.3% headguards.	None
Jeffries et al. (2020)	None	8.7% believed that headgear prevented concussion, 20% believed that mouthguards prevented concussion.	35.4% of players wore headgear.	None
Ruedl et al. (2012a)	80% agreed that helmets protected against head injuries.	None	65% reported wearing a helmet.	None
Taylor et al. (2005)	73% believed that surfers wearing headgear were less prone to injury.	None	1.9% reported using headgear routinely.	None
Tiryaki et al. (2017)	95% believed mouthguards to be protective.	98% believed that mouthguards could prevent dental injuries.	6.3% reported using a mouthguard.	47% suggested using a mouthguard to their players.
Finch et al. (2003)	80% would wear headgear if it prevented injury.	None	91.4% reported not wearing protective headgear.	None
Provance et al. (2012)	87.7% said that safety was the primary reason for wearing a helmet.	None	83% reported wearing a helmet.	None

Most of the studies examining attitudes toward headgear identified a mismatch in attitudes, risk perception and the use of headgear (Pettersen, 2002; Taylor et al., 2005; Ruedl et al., 2012a,b). This meant that in many cases athletes believed that wearing headgear had a protective effect in terms of reducing the number and/or severity of concussions, head injuries and/or orofacial injuries, but that athletes still chose not to wear headgear either because they felt uncomfortable wearing it (Finch et al., 2001; Pettersen, 2002; Braham et al., 2004; Dean and Bundon, 2020) or that the risk of concussion did not apply to them (Taylor et al., 2005). For instance, in Pettersen's (2002) study of Canadian rugby players, 62% believed that wearing headgear could prevent concussion. Despite the players' belief that headgear offered protection against concussion, only a minority reported wearing it (27%) and a few (24%) felt that its use should be compulsory.

A notable exception to this mismatch between attitudes and use of headgear is found in Provance et al. (2012), who examined recreational skiers between 6 and 17 years of age. In this study selection, almost all those wearing a helmet (87.7%) reported that they did it for safety reasons and to prevent head injuries (Provance et al., 2012).

Athletes' use of protective headgear was also associated with an earlier experience of injury or perception of risk (Finch et al., 2003; Kahanov et al., 2005; Inness and Morgan, 2015; Tiryaki et al., 2017; Pratt et al., 2019). Here, athletes who perceived the risk of concussion in their sport as low to moderate wore protective headgear less often (Finch et al., 2003; Taylor et al., 2005). The most frequently mentioned barriers to wearing protective headgear were: “its use is not mandatory” (Pettersen, 2002; Ross et al., 2010), “it is uncomfortable” (Pettersen, 2002; Braham et al., 2004, Finch et al., 2001; Taylor et al., 2005; Provance et al., 2012; Ruedl et al., 2012a; Dean and Bundon, 2020), “it hinders performance” (Taylor et al., 2005; Dean and Bundon, 2020), “it costs too much” (Pettersen, 2002) and that it affects looks/image (Ross et al., 2010; Inness and Morgan, 2015; Dean and Bundon, 2020). The most common reasons for not using a mouthguard were discomfort and difficulty in breathing or talking (Tiryaki et al., 2017).

Some of the studies indicate that athlete demographics such as age, gender and skill-levels could influence athletes' attitudes and subsequent use of protective headgear (Kahanov et al., 2005; Ruedl et al., 2012a; Vriend et al., 2018), meaning that older and more skilled athletes were more unlikely to wear protective headgear than less skilled and younger athletes. Ruedl, Kopp, Rumpold, Holzner, Ledochowski and Burtscher (2012b) findings also indicate that male athletes are less likely to wear a helmet and more likely to engage in risky behaviour during sporting activity than female athletes. The literature on gender differences in risk taking highlights men's increased tendency toward sensation seeking and belief in a positive outcome of the activity compared to women. It also highlights that men and women have different perceptions of the severity of negative outcomes in their risk taking behaviour (Slovic et al., 2004; Harris and Jenkins, 2006). While these findings are valuable and interesting, more research is needed on these aspects, and in particular on how factors other than risk-taking behaviour and gender influence the use of protective headgear.

### Coaches' Attitudes Toward Protective Headgear in Sport

Only a few of the studies examined coaches' attitudes toward protective headgear in sport (see Table 4). In these studies the use of protective equipment among athletes was not associated with coaches' risk perceptions. In their studies, Tiryaki et al. (2017) and Jeffries et al. (2020) identified a disconnection between coaches' risk perceptions and the implementation of concussion-prevention strategies, such as promoting the use of protective headgear in training and competitions. For instance, Jeffries et al. (2020) found that 70% of collegiate women's soccer athletic coaches believed that cervical strengthening programmes would help to prevent concussion, but that only 17% of the coaches included in the sample used such programmes in their training regimes. Jeffries et al. (2020) reported that only 8.76% of the coaches believed that headgear prevented concussion, while 20.74% believed that mouthguards prevented concussion among football (soccer) players. Similarly, only 33% of coaches in Pettersen's (2002) study of Canadian rugby players believed that wearing headgear could prevent concussion. The low percentages reported in Jeffries et al. (2020) and Pettersen (2002) could be due to the phrasing used in the study designs, as there is a notable difference between believing that headgear or mouthguards can prevent concussion and that this type of protective equipment can provide some level of protection against concussion and other head injuries.

Tiryaki et al. (2017) surveyed 53 coaches and 351 players (aged 12–38 years) to examine the prevalence of dental injuries and awareness of mouthguards as protective equipment in basketball. Tiryaki et al. (2017) found that although 98% of the coaches believed that mouthguards could prevent dental injuries, only 47% suggested their use to their players and only 6.3% of the surveyed athletes reported using them.

In other words, similar to the athletes, the coaches in the reviewed literature believed that wearing headgear and mouthguards had a protective effect in reducing the number and/or severity of concussions and other head injuries, but that many did not advise their athletes to use this type of protective equipment in training and competitions (Taylor et al., 2005; Tiryaki et al., 2017; Jeffries et al., 2020).

## Discussion

The findings in this scoping review suggest that there is a discrepancy between attitudes toward and beliefs about the protective effect of headguards and mouthguards, athletes' behaviour when it comes to wearing protective headgear and coaches' behaviour in terms of recommending the use of protective headgear to their athletes (Tiryaki et al., 2017). Although the majority of athletes in most of the reviewed literature believed that headguards and/or mouthguards could protect them against concussion and other head injuries, relatively few of them reported wearing protective headgear unless it was compulsory.

In the following, our discussion of the results of our review is structured in accordance with the guidance for scoping reviews suggested by Munn et al. (2018). Therefore, we first of all focus on the methodology applied in current research (how research on the field is conducted) and secondly on the identified research gaps in the field.

### How Is Research on Athletes' and Coaches' Attitudes Toward Protective Headgear Conducted?

Current research on athletes' and coaches' attitudes toward protective headgear as concussion prevention is mainly conducted using quantitative methods. One of the studies in the review employed quantitative observation (Braham and Finch, 2004), while 14 studies used quantitative questionnaires to collect their empirical data (Ross et al., 2010; Tiryaki et al., 2017; Jeffries et al., 2020). Two of the studies in the review were prospective single cohort studies (Vriend et al., 2018; Pratt et al., 2019). Only one of the studies was based on a qualitative research design (Dean and Bundon, 2020). In terms of methods and analytical approaches, the quantitative articles mainly consist of descriptive statistics and correlational analyses (Finch et al., 2001, 2003; Pettersen, 2002; Braham et al., 2004; Kahanov et al., 2005; Lehl, 2005; Taylor et al., 2005; Ross et al., 2010; Provance et al., 2012; Tiryaki et al., 2017; Jeffries et al., 2020). Some of the quantitative studies employed more rigorous statistical analyses, such as regression analysis (Ruedl et al., 2012b). One qualitative study (Dean and Bundon, 2020) included participant observation and interviews with 12 experienced surfers from Canada to better understand their attitudes toward the use of protective headgear.

The reviewed literature suggests that the current research on athletes' and coaches' attitudes toward protective headgear is conducted on team sports and individual sports in equal measure. Nine studies were conducted with samples consisting of athletes and coaches from team sports, eight with samples of athletes/coaches from individual sports and one included a sample of coaches from both team and individual sports (Lehl, 2005).

An important finding from the reviewed literature is that most of the identified studies of athletes' and coaches' attitudes toward protective headgear are conducted in countries situated in the global north. This means that samples of athletes and coaches from Europe (Inness and Morgan, 2015; Vriend et al., 2018), Australia (Braham et al., 2004; Kahanov et al., 2005), New Zealand (Pratt et al., 2019), and North America (Dean and Bundon, 2020; Jeffries et al., 2020) appear to be over-represented in this research field.

### Research on Athletes' and Coaches' Attitudes Toward Protective Headgear: Identified Knowledge Gaps

There are limitations and gaps in the reviewed literature that can be addressed in future studies to improve the knowledge about athletes' and coaches' attitudes toward protective headgear in sport. A common limitation in the current research is indirect evidence obtained through self-reporting surveys with relatively small non-representative samples from a variety of team and individual sports. Also, most of the reviewed literature focuses exclusively on athletes (both male and female). Of the 18 reviewed studies, only two focus solely on coaches (Lehl, 2005; Jeffries et al., 2020), and only two include athletes and coaches in their sample (Pettersen, 2002; Tiryaki et al., 2017). Based on the findings of this scoping review, future research is needed that engages with athletes as well as coaches and that focuses specifically on coaches' attitudes and behaviour regarding the use of protective headgear in sport. Concretely, research examining issues and questions such as coaches' perceptions of headgear-usage and head injury, or coaches' attitudes and supportive actions toward athletes' actual use of protective headgear, will expand the existing research literature. Lastly, there is a need for future research that takes sport-specific competition rules into account and how they inform coaches' attitudes and behaviour on the use of protective headgear in everyday training situations.

No longitudinal research designs were identified in this review, which is a significant weakness in this research field. Methodologically, the research field is predominately quantitative, although many of the quantitative studies employ relatively weak statistical measures (correlation and descriptive statistics). In future, quantitative studies should aim for a robust regression analysis approach and multilevel analyses. While the methods and statistical measures employed are first and foremost guided by the research question and focus of the study, statistical measures including regression analyses and multilevel analyses explore causations rather than correlations. Therefore, future research is needed that includes these statistical measures, in addition to descriptive statistics.

There was only one qualitative study in the reviewed literature (Dean and Bundon, 2020). More qualitative studies would be useful to the field and provide novel insights into context-specific information about athletes' motivations for wearing (or not wearing) headgear, as well as the psychological and motivational mechanisms behind the use of protective headgear. Hence, there is a need for future research that explores the thoughts and experiences of athletes and coaches in-depth by means of extensive qualitative interviews.

Many of the studies in the reviewed literature include athletes in a variety of different age groups. For instance, the survey of Pratt et al. (2019) included athletes aged between 18 and 65 in the sample, and the study of Ross et al. (2010) included athletes between 18 and 36. This indicates a need for more age-specific research that targets athletes and coaches in different age groups. As Sarmiento et al. (2017) argue, perceptions of health and safety, the importance of sports competition and the influence of coaches may vary significantly between adult-, youth- and child athletes. Some of the studies in this review support this, as findings indicate that younger athletes are more likely to wear protective headgear than older ones (Provance et al., 2012; Ruedl et al., 2012b). Furthermore, none of the studies in the reviewed literature examined variations in attitudes and behaviour in terms of sociocultural factors, such as ethnicity/ethnic background, gender, rural/urban residence or socioeconomic status.

The low number of studies meeting our inclusion criteria (*n* = 18) suggests that despite the increase in knowledge and awareness of head injuries like concussion in athletes and coaches in a wide range of sports (King et al., 2014; Follmer et al., 2020; Tjønndal et al., 2021), there is a need for more knowledge about athletes' and coaches' attitudes toward protective headgear, their wearing of it (athletes) and recommending (or requiring) it (coaches).

In terms of attitudes toward protective headgear, many athletes claim that they would use protective headgear to a greater extent “if it protected them against head injury” (Finch et al., 2003, p. 506). This underlines the need for more accurate knowledge about the function of protective headgear in sport, especially in the light of the conflicting results on the impact of headgear wearers vs. non-wearers (Broglio et al., 2003; Withnall et al., 2005; Mcintosh et al., 2009; Rodowicz et al., 2015; Baron et al., 2020; Tjønndal et al., 2021). As updated knowledge shows a reduction in head injuries when wearing protective headgear (Broglio et al., 2003; Rodowicz et al., 2015; Baron et al., 2020), educational programmes directed toward athletes and coaches may be beneficial in terms of increasing the use of headgear in sport.

### Reflections

Our scoping review identified a small number of studies that met our inclusion criteria on attitudes toward protective headgear and concussion prevention in sport. This small number reflects that this is a field in need of future research, as outlined in our results and discussion. Most of the reviewed literature focused on athletes' attitudes toward protective headgear as a protective measure against concussion and head injury. Only a handful of articles specifically addressed coaches' attitudes and behaviour. While our review attempts to summarise international research on athletes' and coaches' attitudes toward protective headgear in sport, we are limited by our language skills. As we have only searched for literature in English, we may have excluded relevant knowledge from other parts of the world. This might also explain the over-representation of samples from the global north in the reviewed literature.

A limitation of scoping reviews is that they generally do not appraise the quality of evidence included in the review (Munn et al., 2018). Future efforts should explore the feasibility of conducting a systematic review as a next step in understanding attitudes and behaviour about concussion and head injuries among athletes and coaches.

## Conclusion

The purpose of this scoping review was to summarise and analyse the current research on athletes' and coaches' attitudes toward protective headgear in sports. The analysis of the reviewed literature in this scoping review suggests that there is a discrepancy between athletes' and coaches' attitudes toward protective headgear in sport and their behaviour. Based on the analyses of the reviewed literature, this article identifies some of the knowledge gaps in the field and future directions for research.

As Tjønndal et al. (2021) have noted, the debate about protective headgear in sport is often too simplistic and reduced to a question of “to wear or not to wear.” None of the studies in the reviewed literature distinguished between the different types of headgear (beyond headguard/helmet and mouthguard). Additionally, many athletes in the reviewed literature reported that discomfort and image (“looking bad”) were main reasons for not wearing protective headgear. There is a need for multidisciplinary research looking into the design and development of innovative new designs for protective headgear that might lower the barriers experienced by athletes, and thus increasing the chance of more athletes choosing to wear protective headgear regularly. More research examining athletes and coaches' attitudes and behavioural outcomes is also needed to improve the culture of concussion in sport. Such research should incorporate diverse qualitative and quantitative study designs, include athletes and coaches in their samples and include groups from different socioeconomic and ethnic backgrounds.

## Author Contributions

All authors listed have made a substantial, direct and intellectual contribution to the work, and approved it for publication.

## Conflict of Interest

The authors declare that the research was conducted in the absence of any commercial or financial relationships that could be construed as a potential conflict of interest.
